# Practical Data Science for Information Professionals

**DOI:** 10.5195/jmla.2021.1194

**Published:** 2021-04-01

**Authors:** Shannon Compton

**Affiliations:** 1 scompton@baypath.edu, Hatch Learning Commons, Bay Path University, Longmeadow, MA

*Practical Data Science for Information Professionals* is an excellent resource for librarians and information professionals engaged in data management and access who want to delve more deeply into data retrieval and use. David Stuart wrote this book as a follow-up to his first book *Facilitating Access to the Web of Data* (Facet Publishing, 2011). However, the intent of this publication is to address the newer roles of information professionals with regard to achieving better search results by acquiring basic programming skills. Further, the author explores much more thoroughly the idea that librarians are more and more being tasked with data analysis, visualization, and interpretation. The book is divided into eight chapters. The author also includes visual data in the form of figures and tables, which visually convey data he has highlighted in the text. He also includes boxes that give specific examples of information he has presented. The first chapter essentially defines the term “data science” and reviews its rise in popularity and use. He also briefly presents the argument for librarians and information professionals to understand programming and how to use code to create better searches.

The author begins the second chapter, “Little Data, Big Data,” by giving a brief history of the rise of the term ”big data.” He then discusses ways with which we can interact with and manipulate data, such as application programing interfaces (APIs). Stuart ends the chapter with a discussion of data type definitions, such as scientific, government, and business. He also describes how each type of data is different with regard to the data consumer's needs. Chapters 3 and 4 focus on the data science method and data analysis tools, respectively. Chapter 3 presents examples of how to apply the stages of the data science method (i.e., data collection, cleaning, analysis, and visualization in the information profession). Chapter 4 reviews tools that can be used in the data science method. Specifically, the author presents examples of programming languages and software packages that simplify data analysis. These chapters together do an excellent job of presenting a method for conducting data searches and performing analyses of the collected data. In addition, they describe multiple types of tools, analyze their ease of use, and review appropriate applications of the tools with respect to types of data.

The next three chapters are devoted to describing specific techniques that may be employed by information professionals. Each chapter addresses one such technique. Chapter 5, “Clustering and Social Network Analysis,” focuses on the relationships between people and organizations. The purpose of this chapter is to describe multiple types of network analysis methods and provides easy-to-understand examples in the associated box sections. In chapter 6, entitled “Predictions and Forecasts,” Stuart describes why predictions of user behavior are important and presents two statistical methods that can be employed to analyze data (i.e., regression analysis and exponential smoothing). Chapter 7 delves into how librarians and information professionals can analyze and mine text for informative data collection. The author discusses using natural language processing in comparison to keyword or n-gram usage. In the final chapter, “The Future of Data Science and Information Professionals,” Stuart presents his thoughts on the eight challenges faced by data science, provides ten steps for someone wanting to follow the path of a data scientist, and invites the reader to play. The eight challenges range from data literacy to discovery, security, privacy, and management. Stuart also includes issues around algorithm development and accountability as well as cautions against buying into data hype. The thrust of his ten steps to becoming a data scientist is to be savvy, to constantly update your skills, and to advocate for data science usage but with regard to user privacy. Stuart's final message is that the reader of this book should go out and play data discovery and analysis since this book “has only scraped the surface of what is possible for those interested in the possibilities arising from the increasing quantities of data now available” (p. 145).

By the author's own admission, this book is a quick read and not designed to be an in-depth, all-inclusive dissertation on the subject of data science. What it is, however, is a good introduction into the field of data science for those interested in pursuing a career or expanding their current job responsibilities in this field. The main text does assume that the reader has familiarity with some coding concepts, while an appendix at the end goes into a bit more detail for those that are less familiar. The appendix also includes a reading list of programming books. The publication includes a thorough index and a list of references that were used throughout the book. Overall, I would strongly recommend this book for anyone interested in learning about data collection and analysis as it applies to information professionals or librarians in their professional lives.

